# Breaking the silence: the role of forensic dentistry in the identification and prevention of violence against women: a systematic review

**DOI:** 10.3389/fgwh.2026.1745030

**Published:** 2026-03-16

**Authors:** Camille Drouillard, Ana García Navarro

**Affiliations:** Department of Dentistry, Faculty of Health Sciences, Universidad Europea de Valencia, Valencia, España

**Keywords:** abuse (including physical), abused women, dental professional, forensic odontology, maxillofacial injuries, sexual and emotional

## Abstract

Violence against women is an urgent public health and human rights issue. This study evaluated the effectiveness of forensic dentistry in diagnosing, preventing and comprehensively addressing domestic violence, identifying challenges and proposing improvements. A search of PubMed, Scopus and Web of Science was conducted until December 2024 selecting 9 studies including 949 female victims and 1,274 professionals. The most frequent injuries were maxillofacial and dental fractures, and the aggressors were mainly intimate partners. There was evidence of insufficient training of professionals, with moderate knowledge of physical signs and limited knowledge of behavioural indicators. The main barriers were fear of intervention, lack of protocols and the presence of the aggressor during care. Forensic dentistry plays a crucial role, but it is essential to improve professional training and incorporate specific protocols in clinical practice.

## 1 Introduction

Violence against women constitutes one of the most persistent and devastating human rights violations in today's world, in addition to representing a critical public health issue. According to the World Health Organization (WHO), approximately 30% of women worldwide have experienced physical or sexual violence by an intimate partner at some point in their lives (1, 2). This form of violence has profound physical, psychological, and social consequences and requires identification and intervention strategies from various professional disciplines.

Among them, forensic dentistry has emerged as a crucial tool in the detection of signs of abuse, given that a large proportion of physical assaults affect the craniofacial area (3, 4). Several studies have shown that injuries such as maxillofacial fractures, dental avulsions, contusions, and oral lacerations are common among victims of domestic violence (5, 6). In this regard, the forensic dentist holds a privileged position to identify signs of violence that may go unnoticed by other healthcare professionals (7).

The existing body of knowledge has revealed significant advances in describing the injury patterns associated with domestic violence, as well as in recognizing the ethical and legal responsibilities of dentists in identifying and reporting such cases (8, 9). Recent literature has further emphasized the global and socio-cultural dimensions of this phenomenon, highlighting the crucial role of forensic expertise in documenting injuries and supporting judicial processes, even when research primarily focuses on violence against women (10). However, substantial gaps remain. The lack of specific training in violence detection during undergraduate education, unawareness of action protocols in suspected abuse cases, and the scarcity of applied research that integrates behavioral and emotional aspects of victims within the dental context are recognized barriers (11).

Furthermore, there is ongoing controversy regarding the role that dentists should play in these cases. While some authors advocate for an active role in reporting and supporting victims (12), others warn about the legal and ethical risks of intervening without adequate legal grounds or without ensuring the victim's safety (13). The presence of the aggressor during the consultation, fear of retaliation, and lack of communication skills training to address sensitive topics are recurring obstacles that limit dentists' ability to act effectively.

In this context, forensic dentistry must not only focus on the recognition of physical injuries but also on strengthening the clinical, ethical, and legal training of professionals, implementing early detection protocols, and fostering interdisciplinary work with other sectors of health and justice.

The decision to adopt a dual approach in this review—combining the characterization of injury patterns with the assessment of professional training and preparedness—is grounded in their intrinsic interdependence. While the identification of specific craniofacial injury patterns is essential for the early detection and documentation of violence, such knowledge alone is insufficient if dental professionals lack the clinical, ethical, and legal competencies required to act upon these findings. Conversely, evaluating professional education without reference to the types and contexts of injuries encountered limits its practical relevance. By integrating both dimensions, this review aims to provide a more comprehensive and clinically applicable framework that enhances the effectiveness of forensic dentistry in addressing violence against women.

The main objective of this study is to evaluate the effectiveness of forensic dentistry in the diagnosis, prevention, and comprehensive management of domestic violence against women.

## 2 Materials and methods

This systematic review was conducted following the PRISMA guidelines (Preferred Reporting Items for Systematic reviews and Meta-Analyses) (14), ensuring systematic and comprehensive reporting of all research phases.

### 2.1 PEO question

The research question was formulated using the PEO framework:

- P (Population): Women who are potential or confirmed victims of violence.
- E (Exposure): Use of forensic dentistry to diagnose and document signs of violence (such as oral injuries, human bite marks, maxillofacial fractures) and to develop preventive strategies.
- O (Outcomes):
  - O1: Characteristics of female victims and training of healthcare professionals.
  - O2: Diagnostic techniques and tools.
  - O3: Aggressor profile.
  - O4: Limitations and obstacles.

### 2.2 Eligibility criteria

Titles and abstracts were screened, and those meeting the eligibility criteria were selected for full-text analysis. Primary studies had to be observational, randomized controlled trials, prospective or retrospective cohort studies, or case series; involve human subjects with ≥5 participants; be published using the Latin alphabet from 2020 until December 2024. Included patients were women who were potential or confirmed victims of physical or sexual violence. The selected studies focused on forensic dentistry, general dentistry, forensic medicine, and anthropology.

Age range restrictions excluded children, adolescents, and the elderly. Abstracts, case reports, protocols, expert opinions, letters, posters, systematic reviews, meta-analyses, *in vitro* or animal studies, and outdated or irrelevant forensic genetics and pathology research were also excluded.

### 2.3 Sources of information and search strategy

An automated search was conducted in three major databases (PubMed, Scopus, and Web of Science) using keywords such as: “women”, “female”, “physical abuse”, “victims of violence”, “abused woman”, “abused women”, “battered woman”, “battered women”, “neglect”, “domestic violence”, “orofacial trauma”, “dental trauma”, “bite mark*”, “maxillofacial injuries”, “oral injury”, “forensic odontology”, “forensic odontologist”, “forensic dentistry”, “legal medicine”, “medicolegal”, “dental”, “dentist”, “dental professional”, and “knowledge”. These were combined using Boolean operators (AND, OR, NOT) and controlled vocabulary terms (MeSH, Title/Abstract for PubMed; TITLE-ABS-KEY for Scopus) to ensure broad and accurate results.

The PubMed search was conducted using the following query:

((abused women[MeSH Terms) OR (abused woman[MeSH Terms) OR (woman, abused[MeSH Terms) OR (women, abused[MeSH Terms) OR (battered woman[MeSH Terms) OR (“neglect"[Title/Abstract) OR (battered women[MeSH Terms) OR (domestic violence[MeSH Terms) OR (violence, domestic[MeSH Terms) OR (abuse, partner[MeSH Terms) OR (“intimate partner violence"[Title/Abstract)) AND ((maxillofacial injuries[MeSH Terms) OR (maxillofacial injury[MeSH Terms) OR (“oral injury"[Title/Abstract) OR (“oral injuries"[Title/Abstract) OR (“orofacial trauma"[Title/Abstract) OR (legal medicine[MeSH Terms) OR (dentistry, forensic[MeSH Terms) OR (forensic dentistry[MeSH Terms) OR (medicolegal[Title/Abstract) OR (“dentist*"[Title/Abstract) OR (“dental"[Title/Abstract) OR (“dental professional*"[Title/Abstract)) AND ((tools[Title/Abstract) OR (technic*[Title/Abstract) OR (role[Title/Abstract) OR (investigation[Title/Abstract) OR (knowledge[Title/Abstract) OR (limit*[Title/Abstract) OR (screening[Title/Abstract)) NOT (child*) NOT (children) NOT (adolescent*) NOT (Elder) NOT (older) NOT (animal*).

To identify any potentially eligible studies that might have been missed during the initial search, a review of the reference lists in the bibliographies of each selected study was conducted. Duplicate studies were excluded from the review. Additionally, a manual search was performed for one scientific article.

### 2.4 Study selection process

A three-stage selection process was carried out. The selection of studies was performed by two reviewers (CD, AG). In the first stage, titles were screened to eliminate irrelevant publications. In the second stage, abstracts were reviewed and studies were selected based on study type, type of victims (children, women, elderly), information related to diagnosis or prevention, trauma management, and the assessment of healthcare professionals' knowledge and preventive practices. In the third stage, full texts were read, and data were extracted using a pre-designed data collection form to confirm the eligibility of the studies. Disagreements between the reviewers at any stage were resolved through discussion, and when necessary, a third reviewer was consulted.

### 2.5 Data extraction

The following data were extracted from the included studies and organized in a Microsoft Excel 365 spreadsheet: Study data such as authors, title, year of publication, type of study (randomized controlled or not, prospective, retrospective, cross-sectional, longitudinal, etc.). Victim-related information included: sample size, age, and aggressor profile. Injury data included: type of injury (contusions, lacerations, abrasions, fractures, bite marks) and location. Diagnostic evaluation included: type of tools (if any) and diagnostic techniques (observational, radiographic, questionnaire-based). Prevention evaluation included: teaching method (yes or no, and if yes, the type of program used), assessment of professionals' knowledge (recognition of physical signs and behavioural indicators), and use of protocols (yes or no, and if yes, the type of protocol used). Also recorded were limitations and barriers in the use of forensic dentistry for the prevention, diagnosis, and management of domestic violence.

### 2.6 Quality assessment

Risk of bias was assessed by two reviewers (CD, AG) with the aim of analysing the methodological quality of the included articles. For evaluating the quality of qualitative and observational studies, the CASPe checklist “10 questions to help you understand a qualitative study” (15) was used. For the cohort study, the CASPe checklist “11 questions to help you understand a cohort study” (16) was applied. A score of more than 6 “YES” answers was considered indicative of low risk of bias, while a score of 6 or fewer was considered high risk of bias. The STROBE guideline (17) was also used to assess limitations in cross-sectional studies; studies fulfilling all 22 criteria were deemed to have a low risk of bias, whereas failure to meet more than seven criteria indicated a high risk of bias, thus compromising the reliability of the results. Inter-examiner agreement in the quality assessment was measured using Cohen's kappa coefficient, following the scale proposed by Landis and Koch (18).

### 2.7 Data synthesis

In order to summarize and compare outcome variables across the different studies, the means of the main variable values were grouped according to the study group. Since the means reported in the analysed studies came from samples with different numbers of female victims of domestic violence and healthcare professionals involved in knowledge assessments, it was necessary to calculate the weighted mean to obtain representative results. A meta-analysis could not be conducted due to the lack of randomized studies comparing treatment groups; therefore, the findings were presented as a descriptive analysis of the variables.

### 2.8 Study selection

**Figure 1:**
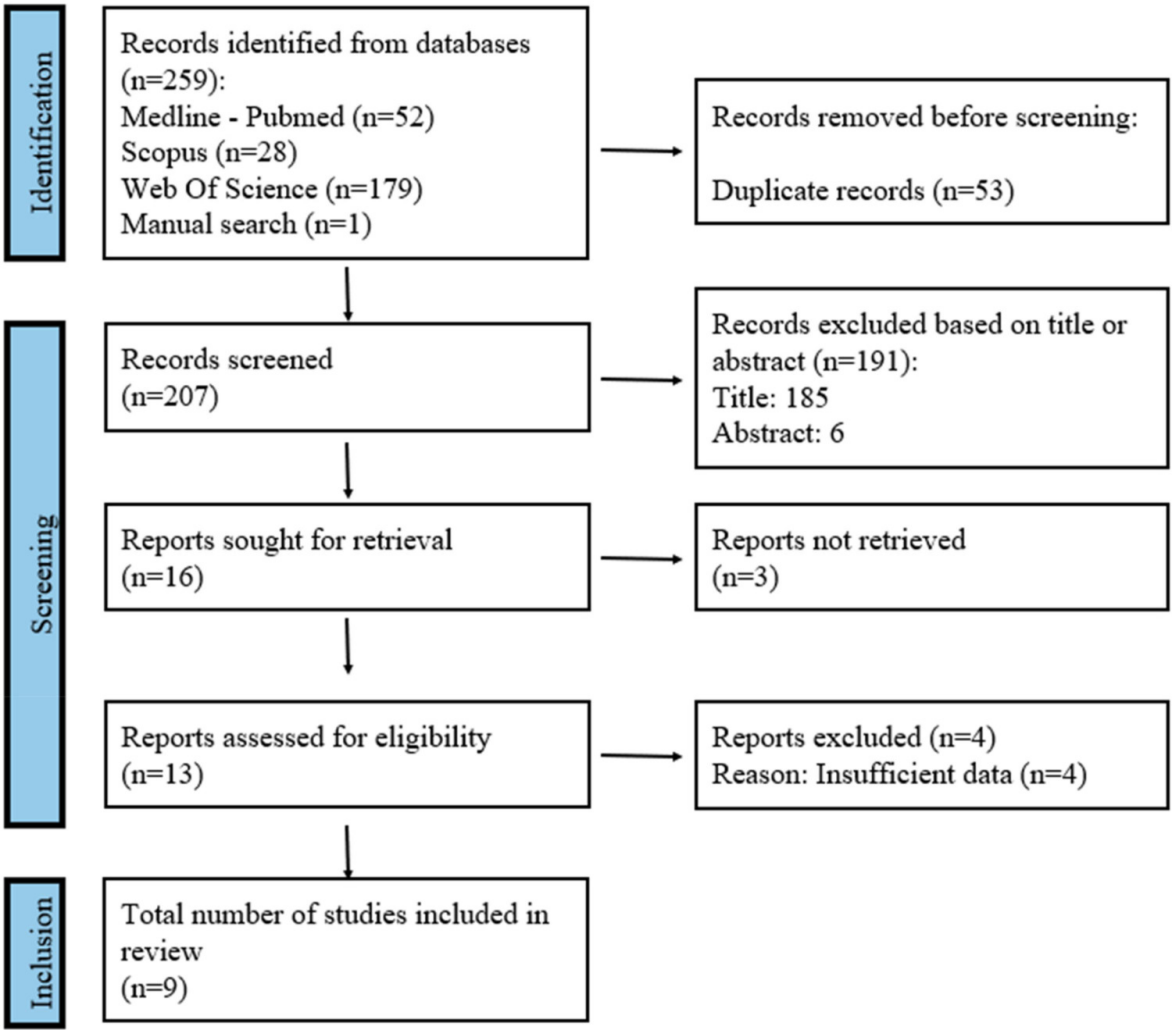
Flow diagram of the search and title selection process during the systematic review.

A total of 259 articles were retrieved from the initial search process: Medline-PubMed (*n* = 52), Scopus (*n* = 28), and Web of Science (*n* = 179). In addition, 1 additional study was identified through manual search. Of these publications, 15 were identified as potentially eligible through title and abstract screening. Full-text articles were subsequently obtained and thoroughly evaluated. As a result, 9 articles met the inclusion criteria and were included in this systematic review (Figure 1). The inter-examiner agreement (k-value) for study inclusion was 1.0 for both title/abstract screening and full-text evaluation, indicating “perfect” agreement according to the criteria of Landis and Koch (18).

## 3 Results

### 3.1 Analysis of the characteristics of the reviewed studies

Of the 9 articles included in the present review, 6 focused on the assessment of knowledge related to domestic violence (19–24), and 3 examined cases of domestic violence against women (25–27).

Six of the articles were cross-sectional studies (19, 20, 23–26), one was a quasi-experimental study (22), one was a qualitative study (21), and one was a cohort study (27).

### 3.2 Methodological quality assessment

The two qualitative and observational studies were considered to have a low risk of bias (Table 1). The cohort study was also classified as having a low risk of bias (Table 2). Among the cross-sectional studies, five were considered to have a high risk of bias, and one was assessed as having a moderate risk of bias (Table 3). The k-value (Cohen's kappa test) for inter-reviewer agreement on methodological quality assessment was 1, indicating “perfect agreement”, according to the scale proposed by Landis & Koch (18).

**Table 1:** Risk of bias assessment for qualitative and observational studies according to the CASPe guide for qualitative research.

CASPe: qualitative study		
	Farmer et al. (21)	Femi-Ajao et al. (22)
1. Were the research objectives clearly defined?	Yes	Yes
2. Is the qualitative methodology appropriate?	Yes	Yes
3. Is the research method suitable for achieving the objectives?	Yes	Yes
4. Is the participant selection strategy consistent with the research question and the method used?	Yes	Yes
5. Are the data collection techniques consistent with the research question and the method used?	Yes	Yes
6. Has the relationship between the researcher and the research subject been reflected upon (reflexivity)?	Partial	Partial
7. Have ethical considerations been taken into account?	Yes	Yes
8. Was the data analysis sufficiently rigorous?	Yes	Yes
9. Are the results clearly presented?	Yes	Yes
10. Are the research findings applicable?	Yes	Yes
Overall risk of bias	LOW	LOW

**Table 2:** Risk of bias assessment of the cohort study according to the CASPe guide for cohort studies.

CASPe: estudio de cohorte	
	Levin et al. (27)
1. Does the study address a clearly focused issue?	Yes
2. Was the cohort recruited in an acceptable way?	Yes
3. Was the exposure accurately measured?	Yes
4. Were confounding factors identified and considered?	Yes
5. Was the follow-up sufficiently long and complete?	Yes
6. What are the results?	Yes
7. How precise are the results?	Partial
8. Do you believe the results?	Yes
9. Do the results fit with other available evidence?	Yes
10. Can the results be applied to the local population?	Yes
11. Will the results change your clinical practice?	Yes
Overall risk of bias	LOW

**Table 3:** Risk of bias assessment of cross-sectional studies according to the STROBE guidelines.

STROBE							
		Alothmani et al. (25)	Alshouibi et al. (19)	BoYes et al. (20)	Gujrathi et al. (26)	Isaila et al. (23)	Meseli et al. (24)
Title and abstract	(1a) Indicate the study design with a commonly used term in the title or abstract.	Yes	Yes	Yes	Yes	Yes	Yes
	(1b) Provide an informative and balanced summary of what has been done and what has been found in the abstract.	Yes	Yes	Yes	Yes	Yes	Yes
Introdution							
Background	2. Explain the scientific background and rationale for the research being reported.	Yes	Yes	Yes	Yes	Yes	Yes
Objectives	3. State the specific objectives, including pre-established hypotheses.	Yes	Yes	Yes	Yes	Yes	Yes
Methods							
Type of study	4. Presentar los elementos clave del tipo de estudio al principio del documento	Yes	Yes	No	Partial	No	Yes
Setting	5. Describe the relevant setting, locations and dates, including recruitment, exposure, monitoring and data collection periods.	Yes	Yes	Yes	Yes	Partial	Partial
Participants	6(a) State the eligibility criteria and the sources and methods of selection of participants.	Yes	Yes	Yes	Partial	Yes	Yes
Variables	7. Clearly define all outcomes, exposures, predictors, potential confounders, and effect modifiers.	Yes	Yes	Yes	Partial	Partial	Partial
Sources of data/measurement	8. For each variable of interest, provide data sources and details of assessment (measurement) methods. Describe the comparability of assessment methods if more than one group is involved.	Yes	Yes	Yes	Yes	Yes	Yes
Bias	9. Describe the efforts made to address potential sources of bias.	Partial	Yes	No	Partial	Partial	Partial
Study size	10. Explain how the study size was arrived at	No	Partial	No	No	No	No
Quantitative variables	11. Explain how quantitative variables were treated in the analyses. If applicable, describe which groupings were chosen and why.	Partial	Yes	No	Partial	Partial	Partial
Statistical methods	(12a) Describe all statistical methods, including those used to control for confounding factors.	Partial	Yes	No	Partial	Partial	Partial
	(12b) Describe the methods used to examine subgroups and interactions.	No	Partial	No	No	No	No
	(12c) Explain how missing data were treated.	No	Partial	No	No	No	No
	(12d) If applicable, describe the analytical methods taking into account the sampling strategy.	No	Partial	No	Partial	No	No
	(12e) Describe any sensitivity analysis.	No	No	No	No	No	No
Results							
Participants	(13a) Report the number of individuals at each phase of the study: number of individuals potentially eligible, screened for eligibility, confirmed eligible, enrolled in the study, completing follow-up and analysed.	Yes	Yes	Yes	Yes	Yes	Yes
	(13b) Indicate the reasons for non-participation in each phase.	No	No	No	No	No	No
	(13c) Consider the use of a flow chart.	N/A	N/A	N/A	N/A	N/A	N/A
Descriptive data	(14a) Indicate characteristics of study participants (e.g., demographic, clinical, social) and information on exposures and potential confounders.	Yes	Yes	Yes	Yes	Yes	Yes
	(14b) Indicate the number of participants with misYesng data for each variable of interest	No	No	No	No	Partial	Partial
Outcome data	15. Report the number of outcomes or summary measures.	Yes	Yes	Yes	Yes	Yes	Yes
Main findings	(16a) Provide unadjusted estimates and, if appropriate, confounder-adjusted estimates and their precision (e.g., 95% confidence interval). Clarify which confounders were adjusted for and why they were included.	Yes	Yes	Partial	Partial	Partial	Partial
	(16b) Indicate the limits of the categories when continuous variables have been categorised.	Yes	Yes	Partial	Partial	Partial	Partial
	(16c) If appropriate, consider translating relative risk estimates into absolute risk for a meaningful time period.	No	No	No	No	No	No
Other analyses	17. Report other analyses performed, e.g., subgroup and interaction analyses and senYestivity analyses.	No	Partial	No	No	No	No
Discussion							
Key results	18. Summarise the main results in relation to the study objectives.	Yes	Yes	Yes	Yes	Yes	Yes
Limitations	19. Discuss the limitations of the study, including sources of potential bias or imprecision. Discuss both the direction and magnitude of any potential bias.	Yes	Yes	Yes	Yes	Partial	Partial
Interpretation	20. Provide a conservative overall interpretation of the results taking into account the objectives, limitations, multiplicity of analyses, results of similar studies, and other relevant evidence.	Yes	Yes	Yes	Yes	Yes	Yes
Generalisability	21. Discuss the generalisability (external validity) of the results of the study.	Yes	Yes	Yes	Partial	Partial	Partial
Other information							
Funding	22. Indicate the source of funding and the role of the funders of the present study and, if applicable, of the original study on which the present article is based.	No	No	No	No	No	No
Overall risk of bias		HIGH	PARTIAL	HIGH	HIGH	HIGH	HIGH

### 3.3 Synthesis of results

#### 3.3.1 Age

**Figure 2:**
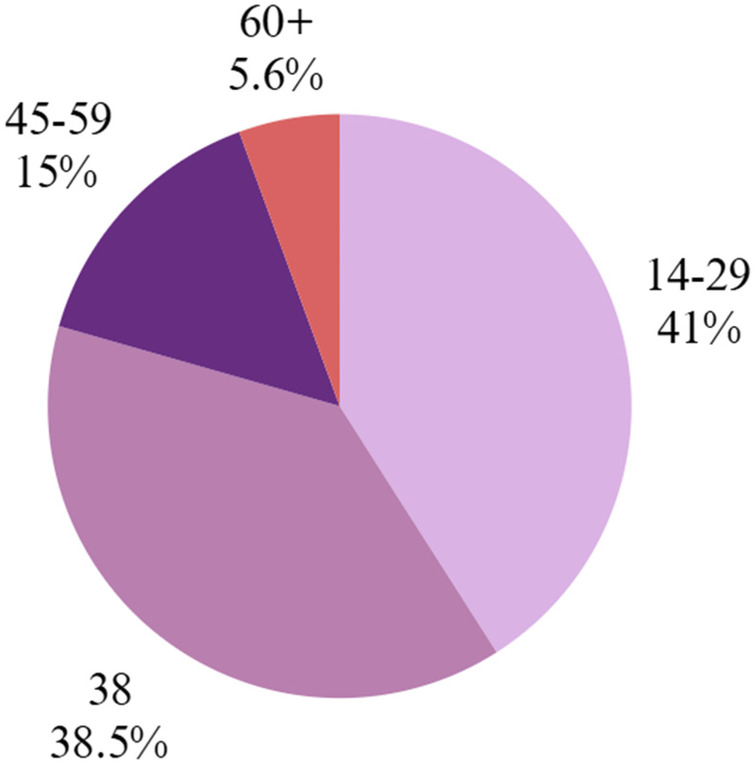
Breakdown of female victims by age group (25–27).

Maxillofacial injuries in 234 female victims of violence were assessed across three studies. Levin et al. (27) analyzed hospitalized women in Israel between 2011 and 2021, reporting a mean age of 44 years. Gujrathi et al. (26) studied 96 patients in the United States with facial injuries resulting from intimate partner violence, with a mean age of 35 years (range 19–76). Alothmani et al. (25) evaluated 100 married women in Saudi Arabia treated for dental trauma, with a mean age of 28.4 ± 5.7 years, ranging from 18 to 60. The overall mean age of participants was 35.8 years. Descriptive results on the age of women victims of violence are shown in Figure 2.

#### 3.3.2 Type of injuries and trauma location

**Figure 3:**
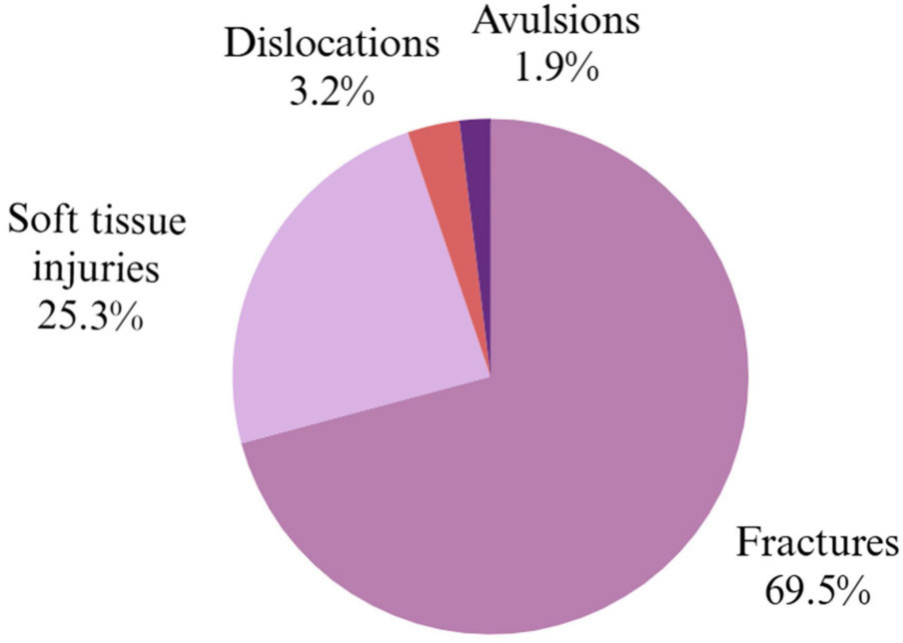
Overall distribution of injury types observed in female victims of domestic violence (25–27).

A total of 290 maxillofacial injuries were documented, including 214 fractures and 78 soft tissue injuries. Only Alothmani et al. (25) reported cases of dental luxations (10) and avulsions (6), with no study mentioning bite marks. Levin et al. (27) identified the most affected areas as the maxilla, zygomatic bone, and mandible, with such injuries accounting for 5% of hospitalizations due to domestic violence. Gujrathi et al. (26) found that 67.7% of victims sustained injuries to the midface, primarily involving the nasal bones, mandible, and orbital bones, with 39.4% presenting soft tissue trauma in the periorbital region. Alothmani et al. (25) focused on dental trauma, reporting mostly crown fractures (84%), followed by luxations (10%) and avulsions (6%), mainly affecting anterior teeth. In all studies, the predominant mechanism of assault was direct impact from physical blows. Descriptive results on the type of injuries, location of trauma and mechanism of aggression are shown in Figure 3.

#### 3.3.3 Education and training of professionals

**Figure 4:**
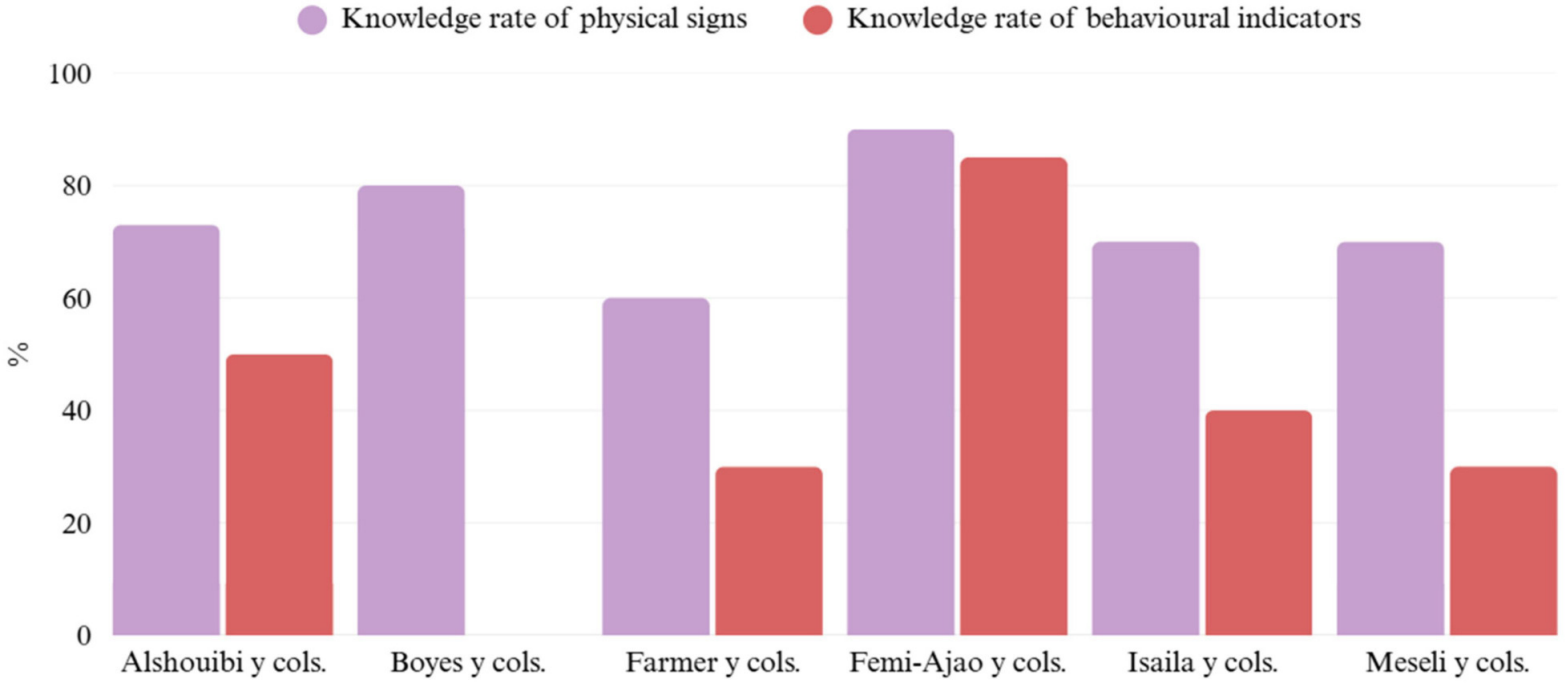
Knowledge rate of physical signs and behavioural indicators by dental professionals (19–24).

A total of 1,274 individuals participated in the studies, including 772 dentists and 437 students. Most had not received prior training in violence detection, except for the study by Femi-Ajao et al. (22), which incorporated training through the IRIS program. The average knowledge level regarding physical signs of interpersonal violence—such as dental fractures, facial injuries, or bruising—was 73.8%, while knowledge of behavioral indicators—such as avoiding eye contact or showing fear—was considerably lower (47%). Additionally, studies by Alshouibi et al. (19), Farmer et al. (21), Femi-Ajao et al. (22), and Isailă et al. (23) revealed a limited level of confidence to intervene in cases of violence, primarily due to the lack of specific training. Boyes et al. (20) also reported that professionals often feel uncomfortable or unsure about how to act due to unfamiliarity with referral procedures. Descriptive results on the assessment of professional knowledge are shown in Figure 4.

#### 3.3.4 Diagnostic techniques and tools

Regarding diagnostic techniques, Levin et al. (27) conducted a retrospective analysis of hospital records from the Israeli National Trauma Registry, without specifying the use of particular imaging modalities. In contrast, Gujrathi et al. (26) employed a systematic radiologic approach using head and facial computed tomography (CT) scans, enabling precise identification of fractures and soft tissue injuries associated with intimate partner violence. Alothmani et al. (25) combined clinical evaluation with periapical radiographs to diagnose dental trauma, also employing pulp vitality tests, palpation, and percussion to determine the type of injury and the need for endodontic treatment.

#### 3.3.5 Aggressor profile

In the reviewed studies, the perpetrator was consistently identified as the intimate partner or the female victim's male spouse. In the study by Alothmani et al. (25), all women confirmed the assault using the HITS screening tool, while Gujrathi et al. (26) and Levin et al. (27) specifically selected cases involving intimate partner violence.

#### 3.3.6 Limitations and barriers

The main barriers to detecting violence included fear of offending the patient, high workload, lack of training, the presence of the aggressor, and ethical concerns regarding confidentiality. A low correlation was also noted between behavioral knowledge and the professional's age. As potential improvements, the studies proposed strengthening continuing education, establishing clear referral protocols, incorporating training at the undergraduate level, and reinforcing ethical education.

## 4 Discussion

### 4.1 Age

The studies by Levin et al. (27), Gujrathi et al. (26), and Alothmani et al. (25) indicate that intimate partner violence primarily affects young adult and middle-aged women, although it also occurs among older women. In this context, the review by Roberto et al. (28) emphasizes that violence does not cease in advanced age but tends to manifest more in psychological rather than physical forms. Research such as that by Regueira-Diéguez et al. (29) in Spain and Saddki et al. (6) in Malaysia confirms that the majority of victims are between 21 and 50 years old. These findings are consistent with the meta-analysis by de Souza Cantão et al. (30), which highlights a high prevalence of oral and maxillofacial injuries among women aged 18–45, revealing a pattern of repeated abuse during an active stage of women's social and reproductive lives.

### 4.2 Type of injuries and trauma location

At both clinical and forensic levels, facial injuries are the most common among victims of domestic violence, with maxillofacial fractures and soft tissue injuries being particularly prevalent. Zeitler (31) identified fractures of the nose, mandible, and orbital bones as frequent, often accompanied by dental trauma. Caldas et al. (32) reported that although perioral soft tissue injuries predominate (80.1%), dental fractures, luxations, and avulsions result in the most severe sequelae. Similarly, Alothmani et al. (25) reported crown fractures in 84% of cases, while Garbin et al. (33) found dental fractures in 59.1% of victims. In a large-scale study, Gassner et al. (34) confirmed a high frequency of dental subluxations (50.6%) and crown fractures (37.5%).

Regarding the affected bone structures, studies by Gujrathi et al. (26), Levin et al. (27), and Saddki et al. (6) highlight the predominance of fractures in the midface, mainly involving the nasal bone, zygomatic bone, and maxilla. Radiologic literature, such as that by Tang et al. (35), supports these findings and emphasizes the diagnostic value of computed tomography. Ochs et al. (36) and Percacciante et al. (37) concluded that injuries to the head, neck, and face are sensitive indicators for identifying partner violence, although they have limited specificity.

Finally, punches and slaps, mostly directed at the face, have been identified as the most frequent mechanisms of assault, causing perioral injuries as well as nasal and dental fractures. Studies such as those by Mgopa et al. (38) and Rubini et al. (39) stress that, although some victims do not exhibit severe visible injuries, they suffer significant functional and psychosocial consequences. The absence of bite marks is also a relevant finding, as their presence may indicate more intimate and severe forms of abuse.

### 4.3 Education and training of professionals

The data analyzed reveal a significant gap in the training of dentists and dental students regarding the detection and management of signs of intimate partner violence—a deficit widely documented in the literature. Bregulla et al. (40) note that, despite the strategic position of dentists, education on domestic violence remains marginal in training programs, leaving many professionals unprepared to act. This lack of training results in uneven knowledge: while 73.8% can identify physical signs, only 47% recognize behavioral indicators. This disparity, also observed by Meseli and Yildiz (24), demonstrates that although visible injuries are recognized, subtle psychological signs—such as avoidance of eye contact or submissive behavior—often go unnoticed.

The low self-confidence to intervene, highlighted by Farmer et al. (21), underscores the need for more structured and practical educational programs. Initiatives such as the DRiDVA program, evaluated by Femi-Ajao et al. (22), have shown that specific training, along with clear protocols and the support of a designated professional figure, significantly improves the knowledge, awareness, and confidence of dental personnel. Despite differences in experience levels, no clear correlation has been found between academic seniority and the ability to address these cases. Many experienced dentists lack specific training, while students, though less clinically experienced, may exhibit greater sensitivity if they have received relevant education. Auxiliary staff also play a key role, though they typically have more limited preparation.

Bregulla et al. (40) and Buchanan et al. (41) agree that targeted training, such as workshops focused on intimate partner violence, increases knowledge and referral capacity among dental students. Similarly, Coulthard et al. (42) demonstrated that programs like IRIS-Dental, which combine practical training, record-keeping tools, and connections to support services, enhance the detection and referral of victims.

Taken together, the studies show that both professionals and students recognize the importance of the dentist's role in addressing domestic violence and emphasize the urgent need to strengthen training—not only in recognizing physical injuries but also in detecting behavioral signs—always within an ethical framework that respects confidentiality and informed consent.

### 4.4 Diagnostic techniques and tools

The results reveal considerable methodological diversity among the analyzed studies. Levin et al. (27) employed a retrospective approach based on hospital records, which is useful for obtaining large-scale epidemiological data but limited in detecting subtle patterns due to the lack of detailed imaging techniques. In contrast, Gujrathi et al. (26) used computed tomography, allowing for a more accurate characterization of injuries associated with intimate partner violence. Avon (8) emphasized the essential role of forensic dentists in identifying interpersonal violence through the comparison of radiographic records, while Jayakrishnan et al. (43) highlighted the value of forensic dentistry in the analysis of hard tissues and anatomical features.

Alothmani et al. (25) demonstrated that it is possible to identify dental trauma related to violence through basic clinical tests and periapical radiographs, even in non-hospital settings. However, Lincoln and Lincoln (44) and Moreira and Pinto da Costa (45) stressed that the detection of violence depends not only on technical tools but also on the willingness to act and knowledge of legal and institutional protocols.

Finally, Pereira et al. (46) and de Jesus Santos Nascimento et al. (47) emphasized the need for a comprehensive approach in emergency dentistry—one that combines technical skills, active listening, and referral protocols—stating that the lack of training and institutional support limits the effective identification of violence cases.

### 4.5 Aggressor profile

The studies reviewed consistently indicate that the perpetrator of documented violence is usually the victim's current intimate partner or male spouse.

This trend, observed in research such as that of Alothmani et al. (25), where all female victims identified their partner as the perpetrator using the HITS test, is consistent with what has been described in other studies, such as those by Gujrathi et al. (26) and Levin et al. (27), which focus specifically on situations of violence perpetrated by romantic partners. These data confirm that interpersonal violence in intimate settings has a strong gender component, which poses significant ethical and clinical challenges in dental practice.

From a forensic dental perspective, Jayakrishnan et al. (43) emphasised that a detailed analysis of orofacial injuries can document abuse and help reconstruct the context of assaults, often correlated with low socioeconomic status, thus providing crucial objective clinical evidence in cases where the perpetrator is close to the victim.

Coulthard et al. (42), as part of the IRIS programme adapted to dental clinics, proposed equipping dental teams with practical tools and referral protocols to ensure a safe and confidential environment for victims. Similarly, Pereira et al. (46) and de Jesus Santos Nascimento et al. (47) emphasised the need for comprehensive and humanised care. They stressed that professionals must go beyond physical treatment, paying attention to emotional or contextual indicators and recognising that the intimate environment, traditionally considered safe, can in fact be a source of violence.

### 4.6 Limitations and barriers

Bregulla et al. (40) and Buchanan et al. (41) agree that although dentists occupy a privileged position to detect signs of violence, the fact that the aggressor is part of the victim's intimate circle often hinders their intervention. The lack of training, legal uncertainty, and unawareness of appropriate protocols lead to reluctance, as also noted by Lincoln and Lincoln (44), who emphasized that the aggressor's power dynamic can extend into the clinical space, intimidating both the victim and the professional. Moreira and Pinto da Costa (45) highlighted that the presence of the aggressor and the lack of confidential spaces are critical barriers that inhibit victims from speaking out.

From a sociological perspective, Moreira and Pinto da Costa (48) emphasized that the domestic setting is the main environment where violence is perpetuated, and intervention becomes especially difficult when the perpetrator is present. Additionally, de Jesus Santos Nascimento (47) and Pereira (49) pointed out the ethical dilemma of when to break confidentiality in order to protect the patient, a decision made more difficult by the lack of clear protocols and legal support.

As potential solutions, the studies propose continuous training in gender-based violence, interdisciplinary education at the undergraduate level, the implementation of referral protocols, and the ethical strengthening of professional approaches. Ultimately, it is emphasized that the role of the dentist is not to resolve conflicts or offer counseling, but rather to recognize signs of violence, intervene prudently, and properly refer victims to appropriate services.

The legal framework governing the reporting of domestic violence by healthcare professionals varies from country to country but is generally based on a balance between medical confidentiality and victim protection. In France, doctors and dentists are bound by professional confidentiality (Art. 226-13 of the Penal Code), with no general obligation to report abuse; however, Article 226-14 of the Penal Code, amended by the Law of 30 July 2020, authorises the lifting of confidentiality in cases of immediate danger to an adult victim under the influence of another person. In Spain, although Organic Law 1/2004 recognises gender-based violence and organises its management, there is no explicit obligation for healthcare professionals to report it automatically, as their role is mainly to issue certificates for evidentiary purposes. More broadly, European legal systems favour regulated and optional reporting mechanisms rather than a strict criminal obligation, highlighting the ethical and legal complexity of medical intervention in cases of domestic violence.

### 4.7 Study limitations

Based on the risk of bias assessment using the STROBE guidelines, the article by Alothmani et al. (25) clearly states its objectives and cross-sectional methods; however, the sample size is small and no formal statistical power calculation is provided (items 10 and 11). The study reports prevalence outcomes with basic statistical analyses but offers a limited description of potential sources of bias (item 9). The retrospective study by Boyes et al. (20), which sampled patients treated at a trauma center, addresses several STROBE items but does not constitute a purely cross-sectional design. While information regarding setting, time frame, and outcomes is provided, the study lacks a formal sample size calculation and includes limited adjustment for confounding factors.

The retrospective observational study by Gujrathi et al. (26) describes the study population, setting, time period, and data sources in considerable detail and discusses its findings in comparison with previous literature. Nevertheless, no sample size calculation or formal confounding analysis is reported. The studies by Alshouibi et al. (19), Isaila et al. (23), and Meseli and Yildiz (24) are explicitly identified as survey-based cross-sectional studies and generally comply with most STROBE criteria. To varying degrees, they lack a formal sample size calculation, detailed statistical analyses, comprehensive assessment of confounders, and clear reporting of funding sources (item 22). Overall, while most studies address the core STROBE components (title/abstract, objectives, methods, results, limitations, and discussion), closer adherence to the STROBE checklist for cross-sectional studies would strengthen their methodological transparency.

Regarding the evaluation of the studies by Farmer et al. (21) and Femi-Ajao et al. (22), both are high-quality qualitative studies with clearly defined objectives, appropriate methodologies (interviews and focus groups), rigorous analytical approaches (thematic or framework analysis), and adequate ethical considerations. Their results are supported by verbatim quotations and include reflections on study limitations. Both studies satisfactorily meet the CASPe criteria for qualitative research, and their findings are highly relevant to domestic violence education within the dental field.

The cohort study by Levin et al. (27) is valid, relevant, and applicable. It presents a low risk of bias in study design and in the measurement of exposures and outcomes. However, more explicit control of confounding variables and greater clarity in the statistical analyses would have strengthened the study.

## Data Availability

The original contributions presented in the study are included in the article/Supplementary Material, further inquiries can be directed to the corresponding authors.
