# Correction: Breaking the silence: the role of forensic dentistry in the identification and prevention of violence against women: a systematic review

**DOI:** 10.3389/fgwh.2026.1837251

**Published:** 2026-04-16

**Authors:** Camille Drouillard, Ana García Navarro

**Affiliations:** Department of Dentistry, Faculty of Health Sciences, Universidad Europea de Valencia, Valencia, España

**Keywords:** abuse (including physical), abused women, dental professional, forensic odontology, maxillofacial injuries, sexual and emotional

The affiliation was incorrectly displayed. It should appear as: Universidad Europea de Valencia, Faculty of Health Sciences, Department of Dentistry, Valencia, España.

